# Electrosprayed Nanoparticles Containing Hydroalcoholic Extract of *Echinacea purpurea* (L.) Moench Stimulates Immune System by Increasing Inflammatory Factors in Male Wistar Rats

**DOI:** 10.34172/apb.2023.031

**Published:** 2022-04-30

**Authors:** Fatemeh Mehdizadeh, Ramin Mohammadzadeh, Hossein Nazemiyeh, Mehran Mesgari-Abbasi, Mohammad Barzegar-Jalali, Morteza Eskandani, Khosro Adibkia

**Affiliations:** ^1^Student Research Committee, Tabriz University of Medical Sciences, Tabriz, Iran.; ^2^Research Center for Pharmaceutical Nanotechnology, Tabriz University of Medical Sciences, Tabriz, Iran.; ^3^Department of Pharmaceutics, Faculty of Pharmacy, Tabriz University of Medical Sciences, Tabriz, Iran.; ^4^Drug Applied Research Center, Tabriz University of Medical Sciences, Tabriz, Iran.

**Keywords:** Electrospray, Echinacea purpurea, Eudragit RS100, Nanoparticles, Immune system

## Abstract

***Purpose:*** *Echinacea purpurea* (L.) Moench is a member of the *Asteraceae* family and is traditionally used mainly due to its immunostimulatory properties. Various compounds including alkylamides and chicoric acid were reported as active ingredients of *E. purpurea*. Here, we aimed to prepare electrosprayed nanoparticles (NPs) containing hydroalcoholic extract of *E. purpurea* using Eudragit RS100 (EP-Eudragit RS100 NPs) to improve the immunomodulatory effects of the extract.

***Methods:*** The EP-Eudragit RS100 NPs with the different extract:polymer ratios and solution concentrations were prepared using the electrospray technique. The size and morphology of the NPs were evaluated using dynamic light scattering (DLS) and field emission-scanning electron microscopy (FE-SEM). To evaluate the immune responses, male Wistar rats were administrated with the prepared EP-Eudragit RS100 NPs and plain extract in the final dose of 30 or 100 mg/kg. The blood samples of the animals were collected and the inflammatory factors and complete blood count (CBC) were investigated.

***Results:*** *In vivo* studies indicated that the plain extract and EP-Eudragit RS100 NPs (100 mg/kg) significantly increased the serum level of tumor necrosis factor-α (TNF-α) and interleukin 1-β (IL1-β) whereas the EP-Eudragit RS100 NPs (30 mg/kg) significantly increased the number of white blood cells (WBCs) compared to the control group. Lymphocytes’ count in all groups was increased significantly compared to the control group (*P*<0.05) whereas other CBC parameters remained unchanged.

***Conclusion:*** The prepared EP-Eudragit RS100 NPs by electrospray technique caused significant reinforcement in the immunostimulatory effects of the extract of *E. purpurea*.

## Introduction

 Nanoparticles (NPs) have been receiving extensive attention due to their unique properties and wide applications in various fields of biology and medicine. The preparation of NPs containing pharmaceutically active substances is the most promising approach for generating new pharmaceutical formulations and also active/passive drug delivery systems (DDSs) to improve the therapeutic effects of drugs and also to reduce off-target toxicity effects.^1-5^ Different types of nanosystems (e.g., polymeric NPs, dendrimers, liposomes, etc.) are used for targeted therapy of various diseases.^6,7^ In this context, several methods including desolvation, dialysis, ionic gelation, nanoprecipitation, solvent evaporation, salting out, supercritical fluid, and spray drying have been developed in a laboratory scale for producing polymeric NPs containing active drugs. Electrospraying is a precious technique that can be used for the formulation of NPs and successful scale-up for the industry.^8,9^ The size and surface of the droplet particles cost-effectively can be controlled in one step at ambient pressure and temperature. This feature makes this method capable of fabricating thin nanofibers to produce DDSs containing ingredients of food, cosmetics, and medicines.^10,11^ Various types of natural and/or synthetic polymers can be used for the preparation of DDSs using the electrospraying technique. The types of polymer and also the preparation process may improve their physicochemical and morphological properties. However, the natural polymers have been more utilized for the preparation of electrosprayed NPs in large part due to their better biocompatibility, lower immunogenicity, adhesion to body tissues, and better clinical performance.^12^ Despite, Eudragit RL and RS are polymers that are broadly used for the production of drugs-NPs with a sustain release profile and special properties such as safety, high permeability, stability, swelling capability in aqueous environments, and solubility in different pHs.^13^

 Medicinal plants as traditional remedies possess important biologically active compounds which comprehensively are used in drug discovery worldwide for the development of lead compounds against different diseases.^14,15^ However, the low solubility of active ingredients in the medicinal plants in aqueous solution limits their usages and pharmacological properties. In this context, vast research has been conducted to prepare physically stable nanosystems containing active phytochemicals including dendrimers, polymers, micelles, and lipid-based NPs using microemulsions possessing.^16^

 The *Asteraceae* is one of the largest plant families, with more than 1620 genera and 23,600 species of plants, shrubs, and trees distributed worldwide. The genus *Echinacea* is one of the most widely used medicinal plants, which is native to North America. The *Echinacea* contains 9 different species of which *E. purpurea*, *E. angustifolia,* and *E. pallida *possess healing properties.^17,18^ *E. purpurea* is a medicinal plant with immune-boosting and anti-inflammatory effects.^19^ Several studies have confirmed the immunomodulatory effects of this species by increasing both innate and specific immunity, anti-inflammatory, antiviral and antimicrobial activity.^20,21^ This plant has been traditionally used for centuries for the treatment of common cold, sore throats, coughs, and other respiratory complications.^18^ Based on the type of extraction and solvents were used during extraction (e.g., aqueous, alcoholic, oily extracts) various compounds were reported with various effects from* E. purpurea*.^22^ Considering the above-mentioned parameters, here we aimed to develop electrosprayed NPs containing extract of *E. purpurea* using Eudragit RS100 (EP-Eudragit RS100 NPs) to improve the pharmacological effect of *E. purpurea*. To this end, EP-Eudragit RS100 NPswere formulated by the electrospray technique with different extract:polymer ratios at various solution concentrations. The morphological and physicochemical feature of the prepared NPs were investigated. Furthermore, the immunomodulatory effects of the prepared EP-Eudragit RS100 NPs in male Wistar rats were assessed and compared with the plain extracts.

## Materials and Methods

###  Materials

*Echinacea purpurea* was purchased from Shafapazhoohan (Tabriz, Iran), ketamine was from Sigma-Aldrich (Diegem, Belgium) and xylazine was purchased from Alfasan (The Netherlands). Tumor necrosis factor-α (TNF-α) kit and interleukin 1-β (IL1-β) kit were purchased from Shanghai Crystal Day Biotech Co. (Shanghai, China). Eudragit® RS100, n-Hexane and methanol were from Merck (Darmstadt, Germany). All other chemical materials were in analytical grade and purchased from Dr. Mojallali Industrial Chemical Complex (Tehran, Iran).

###  Methods

####  Extraction

 In this study, the hydroalcoholic extract (water/ethanol ratio 30:70 v/v) was prepared by the maceration method. The extraction procedure was conducted for at least 24 hours and repeated 3 times. The obtained extract was filtered using filter paper and concentrated by rotary evaporator apparatus at 45°C under vacuum. The obtained powdered extract was dissolved in methanol, and the extraction procedure was continued for 5 hours on a stirrer. Finally, the product was centrifuged (300 g for 30 minutes) and the supernatant was separated for future work.

####  Electrospraying procedure

 A custom-designed electrospray evaporative cooling (ESEC) apparatus (Fanavaran Nano-Meghyas, Tehran, Iran) was operated to formulate EP-Eudragit RS100 NPs. Briefly, the hydroalcoholic extract of *E. purpurea *andEudrRS100 were mixed (1:5 and 1:10; extract: polymer) and dissolved in methanol at the final concentrations of 10, 15, and 20 % (w/v) and ambient temperature (25°C). The liquid jet of the formulated solution was made by utilizing a voltage of 25 kV applied to the syringe tip (gauge 29) connected to a polyethylene-made ring-shaped capillary tube with the inner diameters of 0.1 mm. The prepared solutions were flowed towards a grounded polytetrafluoroethylene coated aluminum, as a collector screen to formed EP-Eudragit RS100 NPs. The distance between the nozzle tip and injection rate was fixed at 20 cm and 2.5 mL/h, respectively.

####  Dynamic light scattering (DLS)

 The size distribution and mean diameter of the electrosprayed NPs were measured by Malvern ZetaSizer NanoSeries (Malvern Instruments, UK).

####  Field emission scanning electron microscopy (FE-SEM)

 The morphology of the prepared NPs was evaluated using a MIRA3 field emission scanning electron microscope (FE-SEM) (Tescan; Brno, Czech) operating at 15 kV. Before evaluation by FE-SEM, the electrosprayed formulations were coated with a thin gold film (about 150 Å in thickness) using gold sputtering apparatus (Emitech K550, Kent, UK).

###  Animal studies

####  Animals

 A total of 40 male Wistar rats (200-250 g) were supplied by the animal center laboratory, Pasteur Institute, Iran. The animals were housed under specific conditions of a 12-12 hours light to dark cycle in an air-conditioned room at 22 ± 2°C with a relative humidity of 50 ± 10%. Standard diet for rats (Behparvar Co., Karaj, Iran) and water were supplied ad libitum. All animal procedures were performed according to the ‘Guide for the Care and Use of Laboratory Animals’ for Laboratory Animal of Tabriz University of Medical Sciences which was in accordance with the National Institutes of Health guidelines (revised 2011) and was approved by the local authorities of animal ethics committees (AEC reference number: TBZMED.VCR.REC.1397.303).

###  In vivo procedure

 In this study, 40 male rats weighing 200 to 250 g were used. The study was performed on five groups each containing eight male rats. In group 1 which was considered the control group, 1 mL of distilled water was gavaged. Groups 2 and 3 received *E. purpurea* extract at 30 mg/kg BW and 100 mg/kg BW, respectively. Groups 4 and 5 received electrosprayed EP-Eudragit RS100 NPs (extract:polymer ratio of 1:5 and final concentration of 10% (w/v)). In all groups, the intervention was done every other day for one month.

###  Measurement of TNF-α and IL1-β

 To evaluate the possible effect of prepared NPs and plain extract of *E. purpurea* on the immune system, the animals were subjected to deep intraperitoneal anesthesia with a mixture of ketamine and xylazine at a dose of 10/60 mg/kg. The blood samples were taken by cardiac puncture. Some of the obtained blood was collected in vials containing anticoagulant ethylenediaminetetraacetic acid (EDTA) and complete blood count (CBC) was analyzed using a Technicon Cell Counter H1 (Hobro, Denmark). The remaining blood samples were kept in the tubes without anticoagulant at laboratory temperature (24 ± 2°C) for 20 minutes for clotting. The serum of coagulated samples was isolated by centrifugation (300 g for 10 minutes) and stored at -70°C until used. The concentration of TNF-α and IL1-β levels in serum samples were quantified by the kits according to the protocols provided by the manufacturer using the ELISA method.

###  Statistical analysis

 Mann–Whitney U and Fisher’s exact tests were applied to compare the groups. Statistical analyses were implemented using SigmaPlot V12 and any variations among the groups were assumed significant at *P* < 0.05 levels. The *in vivo* data were asserted as mean ± SEM and attained from 10 experimented mice. Also, statistical analyses of the mean number and percentage of monocytes, neutrophils, erythrocyte count, hemoglobin, mean MCV, MCH, and MCHC were accomplished using the Tukey post hoc test (*P* < 0.05).

## Results and Discussion

###  The morphology and particle size of electrosprayed EP-Eudragit RS100 NPs 

**Figure 1:**
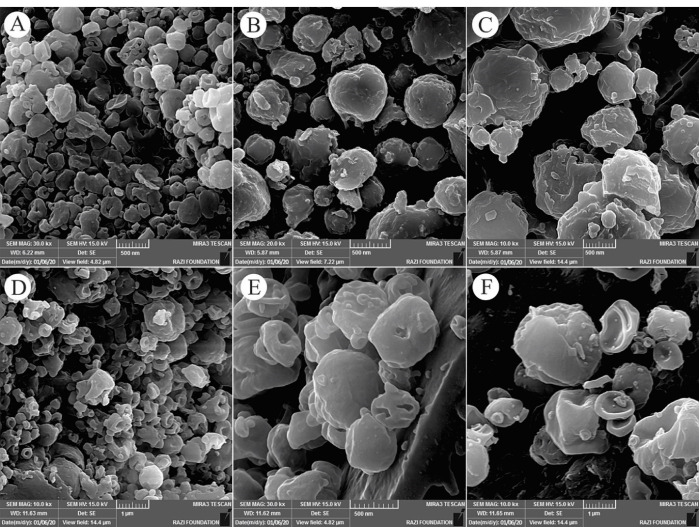
The FE-SEM images of the electrosprayed EP-Eudragit RS100 NPs with extract:polymer ratios and concentrations of A) F1 (1:5, 10%) B) F2 (1:5, 15%) C) F3 (1:5, 20%) D) F4 (1:10, 10%) E) F5 (1:10, 15%) and F) F6 (1:10, 20%).

**Figure 2:**
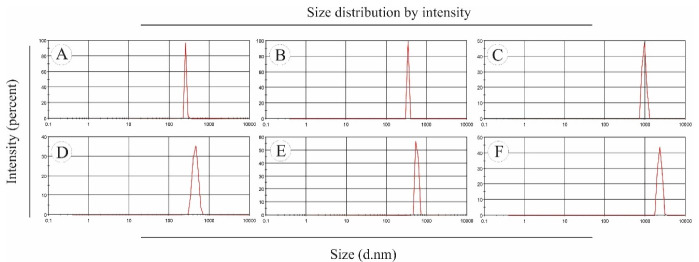
The size distribution of the electrosprayed EP-Eudragit RS100 NPs with extract:polymer ratios and concentrations of A) F1 (1:5, 10%) B) F2 (1:5, 15%) C) F3 (1:5, 20%) D) F4 (1:10, 10%) E) F5 (1:10, 15%) and F) F6 (1:10, 20%).

 The morphology and size of electrosprayed NPs play an important role in their physicochemical properties. In the electrospray method, the size distribution of NPs is affected by various parameters such as type and concentration of polymer, type of solvent and its evaporation rate, polymer diffusion, etc. Also, the flow rate of the polymer solution depends on the nozzle distance from the collector plate.^23,24^ To prepare electrosprayed EP-Eudragit RS100 NPs, the air-dried powdered extract of *E. purpurea* was combined with Eudragit RS100 polymer with the ratios of (1:5) and (1:10) (extract:polymer) and dissolved in methanol at the final concentrations of 10%, 15%, and 20% w/v. The average size and morphology of the prepared NPs were measured using DLS and SEM analyses, respectively after 1:20 dilution with deionized water. The SEM images showed that the prepared NPs were spherical with smooth edges. Besides, DLS results demonstrated that the size of the optimized NPs was 256.1 ± 6.68. The formulations parameters and the mean diameter of the prepared NPs are summarized in Table 1. Besides, Figure 1 shows the morphology of the electrosprayed EP-Eudragit RS100 NPs taken with SEM analyses. Moreover, Figure 2 shows the size distribution of the electrosprayed EP-Eudragit RS100 NPs. In this work, according to the results obtained from DLS and FE-SEM, the size of the spherical with smooth surface NPs in F1 formulation with a drug:polymer ratio of 1:5 and a concentration of 10% (w/v) was 256.1 nm.

**Table 1:** EP-Eudragit RS100 NPs prepared by electrospraying

**Formulation**	**Total concentration (w/v)%**	**The ratio of extract: polymer**	**Particle size±SD (nm)**
F1	10%	1:5	256.1 ± 6.68
F2	15%	1:5	450.5 ± 66.9
F3	20%	1:5	938.6 ± 97.5
F4	10%	1:10	342.0 ± 6.6
F5	15%	1:10	567.4 ± 41.58
F6	20%	1:10	2317 ± 261.1

 During the electrospray process, all influencing factors were kept constant except the concentration and extract:polymer ratio. The concentration of electrospray solution played an important role in the size and morphology of NPs so that at low concentrations of polymer solution, owing to the high surface tension that overcomes the viscoelastic forces, smaller particles are formed. We found that with the increasing of the polymer concentration, the high viscoelastic forces overcame the surface tension and therefore the larger particles are formed. By comparing formulations with similar concentrations, the average particle size in formulations F1, F2, and F3 with an extract:polymer ratio of 1:5 was smaller than the average particle size of the formulations F4, F5, and F6 where the extract:polymer ratio was 1:10. It seems this could be due to reduced electrical conductivity at high concentrations. Similar results were reported for NPs prepared from carbamazepine with PVP K30 polymer, triamcinolone with RS100 eraser polymer, and modafinil with RS100 eraser polymer.^25^ It should be noted that the solvent used should be selected in such a way that as a common solvent, it dissolves both the drug and the polymer. For this purpose, methanol was chosen to dissolve both the extract and the polymer as a common solvent.

###  CBC analyses

 The male rats were administrated daily with the plain extracts of *E. purpurea *(30 and 100 mg/kg) andelectrosprayed EP-Eudragit RS100 NPs (30 and 100 mg/kg), and CBC, as well as serum TNF-α and IL-1β, were analyzed. Table 2 summarizes various components and features of the blood and also the TNF-α and IL-1β serum levels of the animals administrated with the plain extract and EP-Eudragit RS100 NPs. The effects of EP-Eudragit RS100 NPsand plain extract of *E. purpurea* on total WBCs showed that gavage of the NPs containing the extract at a dose of 30 mg/kg caused a significant increase (*P* < 0.05) in white blood cell (WBC) levels. However, the plain crude extract with the same dose could not significantly increase the number of WBCs compared to the control group. The results show that the EP-Eudragit RS100 NPs have better effects than the plain extract. It had been previously shown that curcumin encapsulation by electrospray method in poly-lactic acid polymer leads to longer release and better antibacterial effects of curcumin.^26^ According to the results of the present study and previous studies, it seems that the electrospray method is one of the most cost-effective and efficient techniques for the preparation of polymer NPs that encapsulate high-efficiency hydrophilic and hydrophobic drugs in micro-and nano-capsules.

**Table 2:** Various components of the blood of the animals were administrated with the plain extract of *E. purpurea * and EP-Eudragit RS100 NPs

**Variables**	**Groups**				
	**Control**	**Plain extract of** *** E. purpurea*** **(30 mg/kg)**	**Plain extract of** *** E. purpurea*** **(100 mg/kg)**	***F1*** **(30 mg/kg)**	***F1*** **(100 mg/kg)**
WBC ( × 10^3^/μL)	2.96 ± 0.40	4.67 ± 0.56	4.48 ± 0.30	4.99 ± 0.50	4.73 ± 0.86
Lymph ( × 10^3^/μL)	1.48 ± 0.23	3.4 ± 0.50	3.01 ± 0.17	3.18 ± 0.32	3.23 ± 0.59
Lymph (%)	54.51 ± 3.15	69.15 ± 2.67	67.91 ± 3.11	63.88 ± 3.37	68.15 ± 2.69
Mono ( × 10^3^/μL)	0.59 ± 0.06	0.83 ± 0.13	0.81 ± 0.09	1.11 ± 0.20	0.89 ± 0.28
Mono (%)	22.9 ± 1.69	17.03 ± 0.68	18.03 ± 1.45	22 ± 2.32	18 ± 2.61
Neut ( × 10^3^/μL)	0.47 ± 0.11	0.45 ± 0.02	0.49 ± 0.10	0.62 ± 0.07	0.45 ± 0.01
Neut (%)	16.98 ± 1.79	10.56 ± 1.88	10.66 ± 1.51	12.6 ± 1.04	10.42 ± 1.50
Eos ( × 10^3^/μL)	0.04 ± 0.02	0.03 ± 0.01	0.02 ± 0.00	0.02 ± 0.01	0.05 ± 0.01
Eos (%)	1.51 ± 0.40	0.95 ± 0.22	0.51 ± 0.06	0.46 ± 0.08	1.42 ± 0.37
Baso ( × 10^3^/μL)	0 ± 0.00	0.01 ± 0.00	0.01 ± 0.00	0.01 ± 0.00	0.02 ± 0.01
Baso (%)	0.15 ± 0.03	0.18 ± 0.03	0.18 ± 0.03	0.18 ± 0.05	0.36 ± 0.13
RBC ( × 10^3^/μL)	7.56 ± 0.23	7.77 ± 0.19	7.31 ± 0.23	7.85 ± 0.19	7.11 ± 0.14
HGB (g/dL)	12.91 ± 0.47	14.01 ± 0.40	12.85 ± 0.22	13.16 ± 0.19	12.1 ± 0.33
HCT (%)	44.11 ± 2.04	45.00 ± 1.16	41.85 ± 0.89	44.96 ± 0.64	41.12 ± 0.75
MCV (fl)	58.26 ± 0.90	57.85 ± 0.34	57.3 ± 0.75	57.3 ± 0.86	57.77 ± 0.30
MCH (pg)	17.06 ± 0.35	18.01 ± 0.11	17.6 ± 0.30	16.78 ± 0.28	17.02 ± 0.63
MCHC (g/dL)	29.31 ± 0.63	31.13 ± 0.25	30.71 ± 0.25	29.22 ± 0.32	29.47 ± 0.99

All values are given as mean ± SE in each group. TNF-α: tumor necrosis factor-alpha, IL-1β: Interleukin 1 beta, WBC: white blood cell count, Lymph: Lymphocyte, Mono: monocyte, Neut: neutrophil, Eos: eosinophil, Baso: basophil, RBC: red blood cell count, HGB: hemoglobin, HCT: hematocrit, MCV: mean corpuscular volume, MCH: mean corpuscular hemoglobin, MCHC: mean corpuscular hemoglobin concentration. F1 formulation represents EP-Eudragit RS100 NPs which were spray-dried using the mixture of extract:polymer (1:5) and dissolved in methanol at the final concentration of 10% w/v. F2 formulation represents EP-Eudragit RS100 NPs which were spray dried using the mixture of extract:polymer (1:5) and dissolved in methanol at the final concentration of 15% w/v.

 Another study declared that the hydro-alcoholic extract of *E. purpurea* boosted and stimulated the immune system at different concentrations by increasing WBCs, the average number of lymphocytes, and the amount of phagocytosis, although this effect was greater at lower concentrations.^27^ Administration of EP-Eudragit RS100 NPs (30 mg/kg) could significantly (*P* < 0.05) increase the number of WBCs, but at a dose of 100 mg/kg, no significant change was observed compared to the control group. Statistical analyses of the quantity and percentage of lymphocytes showed a significant increase compared to the control group (*P* < 0.05). *In vitro* and *in vivo* studies have shown that the plain extract of *E. purpurea* stimulates macrophage activity, increases interferon levels, phagocytosis, and cellular respiration. Moreover, it activates lymphocytes by increasing TNF-α, IL-1 and interferon beta (IFN-β), and stimulates the immune system.^28,29^ Statistical analyses of the mean number and percentage of monocytes, neutrophils, erythrocyte count, hemoglobin, mean MCV, MCH and MCHC using Tukey post hoc test (*P* < 0.05) showed that there was no significant change in the treated and control groups. The results of this study showed that EP-Eudragit RS100 NPs increased the number of whole WBCs compared to the plain crude extract of *E. purpurea* in different concentrations.

###  Measurement of TNF-α and IL1-β

**Figure 3:**
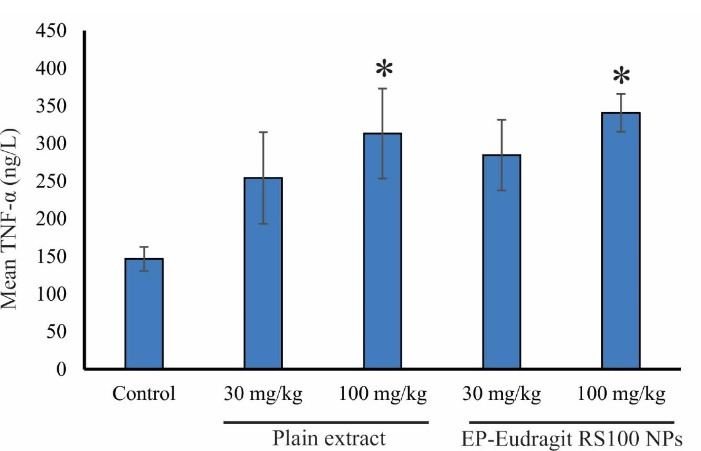
The effects of EP-Eudragit RS100 NPsand the plain extract of *E. purpurea* on TNF-α serum levels in the studied animals. The asterisks indicate a significant difference between control and treatment groups (* the statistical significance was *P* < 0.05).

**Figure 4:**
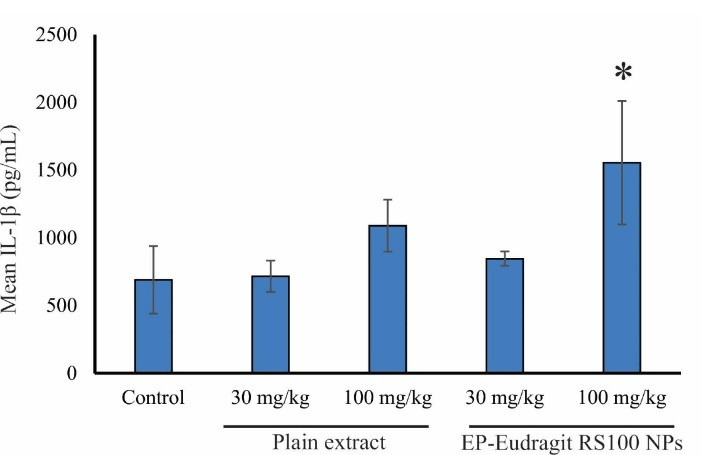
The effects of EP-Eudragit RS100 NPsand the plain extract of *E. purpurea* on the serum levels of IL-1β in the studied animals. The asterisks indicate a significant difference between control and treatment groups (* the statistical significance was *P* < 0.05).

 In the present study, the immunomodulatory effect of EP-Eudragit RS100 NPswas investigated on male rats by evaluating the variations of inflammatory factors and the overall assessment of the various components of the blood using CBC analyses. According to the results, the groups receiving EP-Eudragit RS100 NPswith an extract to polymer ratio of 1:5 and a concentration of 10% (w/v) (F1) and the plain extract of *E. purpurea* at a dose of 100 mg/kg displayed a significant increase (*P* < 0.05) in TNF-α serum levels compared to the control group. This indicates that the plain extract of *E. purpurea* with and without carrier causes a significant increase in TNF-α serum levels. Statistical analysis of IL-1β serum level also showed that administration of EP-Eudragit RS100 NPsat a dose of 100 mg/kg caused a significant increase (*P* < 0.05) in IL-1β serum levels compared to the control group. However, the plain extract of *E. purpurea* at the same dose could not significantly increase the serum levels of IL-1β. Administration of EP-Eudragit RS100 NPsincreased the serum level of IL-1β at higher levels compared to the plain extract of the *E. purpurea* indicating that electrosprayed NPs of *E. purpurea* extract can increase the bioavailability of the plant extract. Studies have shown that NPs act as effective and selective DDSs. They improve the pharmacokinetics of cargos and increase their efficacy and bioavailability. This is due to the rapid endocytosis of the intestinal mucosa and other biological membranes due to the high surface-to-volume ratio of the NPs.^30^ Figure 3 shows the TNF-α serum levels of the animals administrated with the plain extract and EP-Eudragit RS100 NPs. Gavage of EP-Eudragit RS100 NPsand the plain extract *E. purpurea* at a dose of 100 mg/kg caused a significant increase in TNF-α serum level compared to the control group (*P* < 0.05). Administration of EP-Eudragit RS100 NPsat similar doses of 30 and 100 mg/kg also increased TNF-α serum levels but no significant change was seen between groups (*P* > 0.05). Figure 4 shows IL-1β serum levels in the study groups. Gavage of EP-Eudragit RS100 NPsand the plain extract *E. purpurea* at a dose of 100 mg/kg caused a significant increase in serum IL-1β level compared to the control group (*P* < 0.05).

## Conclusion

 Here in this study for the first time, the hydroalcoholic extract of*E. purpurea* was formulated in EudrRS100 polymer using the electrospray method to obtain EP-Eudragit RS100 NPs. The morphology and size of the prepared NPs were investigated and the immunomodulatory properties of the prepared NPs containing the extract were compared with the plain extract. The morphology of the prepared NPs was closely related to parameters such as the extract:polymer ratio, solution concentration, solution flow rate, and nozzle distance from the collecting plate. With increasing polymer concentration as well as extract:polymer ratio, the particle size increased significantly. Both the EP-Eudragit RS100 NPs and the plain extract of *E. purpurea* at a dose of 100 mg/kg significantly increased the serum level of TNF-α compared to the control group in male rats. The gavage of EP-Eudragit RS100 NPs at a dose of 100 mg/kg in male rats significantly increased the serum level of IL-1β compared to the control group. Also, the gavage of EP-Eudragit RS100 NPs at a dose of 30 mg/kg significantly increased the total white blood cell count in male rats. Moreover, the gavage of the plain crude extract of *E. purpurea* and EP-Eudragit RS100 NPs at both doses of 100 and 30 mg/kg caused a significant increase in the number of lymphocytes in the blood. Finally, it can be stated that the electrospray method is a simple, adjustable, efficient, cost-effective, surfactant-free, and industrializable process for the preparation of Eudragit RS100 NPs containing the hydroalcoholic extract of *E. purpurea* to enhance the biological activity of *E. purpurea* (L.) Moench.

## Acknowledgments

 This article is based on a thesis submitted for a Pharm.D degree (No. 4095) in the Faculty of Pharmacy, Tabriz University of Medical Sciences, Tabriz, Iran.

 The authors would like to thank the Research Center for Pharmaceutical Nanotechnology, Tabriz University of Medical Sciences, Tabriz, Iran for their financial support.

## Competing Interests

 The authors report no conflict of interests. The publication has been approved by all co-authors and the responsible authorities at the institute(s) where the work has been carried out.

## Ethical Approval

 All the animal experimental procedures were conducted according to the general guidelines of the Animal Ethics Committee of Tabriz University of Medical Sciences (TBZMED.VCR.REC.1397.303) and carried out in accordance with the National Institutes of Health guide for the care and use of laboratory animals (NIH Publications No. 8023, revised 1978).
